# Effects of a Motivational Climate on Psychological Needs Satisfaction, Motivation and Commitment in Teen Handball Players

**DOI:** 10.3390/ijerph16152702

**Published:** 2019-07-29

**Authors:** Marianna Alesi, Manuel Gómez-López, Carla Chicau Borrego, Diogo Monteiro, Antonio Granero-Gallegos

**Affiliations:** 1 Department of Psychological, Pedagogical and Education Sciences, University of Palermo, 90133 Palermo, Italy; 2 Department of Physical Activity and Sport, Faculty of Sport Sciences, University of Murcia, Santiago de la Ribera, 30720 Murcia, Spain; 3 Sport Sciences School of Rio Maior, Polytechnic Institute of Santarém Research Center in Life Quality (CIEQV), 2040-413 Rio Maior, Portugal; 4 Sport Sciences School of Rio Maior, Polytechnic Institute of Santarém Research Center in Sport, Health and Human Development (CIDESD), 2040-413 Rio Maior, Portugal; 5 Department of Education, Faculty of Education Sciences, University of Almeria, 04120 Almeria, Spain; 6 Health Research Center, University of Almeria, 04120 Almeria, Spain

**Keywords:** Coach, self-determined motivation, team sports, sports adherence, performance

## Abstract

The aim of this study was to examine the effects of the motivational climate created by the coach and perceived by a group of young high-performance handball players on their sport motivation, self-determination, sport psychological needs and sport commitment. The study participants were 479 young handball players. The age range was 16–17 years old. Players were administered a battery composed of a Perceived Motivational Climate in Sport Questionnaire, Sport Motivation Scale, the Basic Psychological Needs in Exercise Scale and Sport Commitment Questionnaire to measure the above-mentioned theoretical constructs. Results showed that the handball players showed high levels of a task-involving climate, of basic psychological needs satisfaction and of self-determined motivation and commitment. Higher levels of basic psychological needs such as autonomy and competence were associated with a higher task-involving climate, self-determined index and sport commitment (task-involving climate–basic psychological needs (β = 0.55; 95% IC 0.387/0.682; *p* = 0.001); Ego-involving climate–basic psychological needs (β = 0.06; 95% IC −0.069/0.181; *p* = 0.387); Basic psychological needs–self-determined index (β = 0.48; 95% IC 0.376/0.571; *p* = 0.001); Self-determined index–commitment (β = 0.58; 95% IC 0.488/0.663; *p* = 0.001). The obtained model showed that basic psychological needs mediated the association between a task-involving climate and self-determination, and self-determination mediated the association between basic psychological needs satisfaction and commitment.

## 1. Introduction

Following the Self-Determination Theory (SDT) [1] and the Achievement Goal Theory (AGT) [2,3], much research has underlined psychological components as a fundamental condition that contributes to athletes’ performance. Sport success is the result of a complex interplay between an athlete’s technical skills, physical condition and adaptive motivational disposition. In detail, this disposition is composed of positive self-esteem and perception of competence, effort attribution style, fitting representation of own abilities, task-oriented goals and persistence when faced with difficulties. All these factors enhance the probability of sport success performance by acting simultaneously to predispose the athlete for a maximum show of personal sport skills [4].

SDT is one of the most used perspectives to investigate life-long motivation in educational settings such as school and sport [1,5,6,7]. On the whole, six forms of motivation varying on regulation and self-determination degree were postulated. These move along a continuum from intrinsic motivation to amotivation and are labelled: intrinsic regulation, integration, identification, introjection, external regulation and amotivation. They fall into two main categories: autonomous (intrinsic, integrated and identified motivation) and controlled (introjected and external motivation) motivation.

Intrinsic motivation concerns behaviors performed for personal aims such as enjoyment, curiosity, satisfaction and interest; integrated regulation concerns behaviors that are aligned with an individual’s self and are congruent with their value system; identified regulation concerns behaviors that are not attractive for themselves but are recognized for their underlying benefits. Introjected motivation refers to behaviors that are moved by external sources that have been internalized; external regulation refers to behaviors that are moved by external causes. Finally, amotivation refers to behaviors characterized by a lack of intention to behave or an interest in the activity.

Autonomy, with competence and relatedness, is conceived as an inherent psychological need that necessitates support from environmental conditions and interpersonal relationships [8,9]. On the whole, autonomous forms of motivation are moved by internal sources and behaviors are perceived as self-authored. They are correlated to a higher level of psychological well-being, more positive affective disposition, improved performance, especially on complex and heuristic activities, and greater persistence in sporting activities [10]. Conversely, controlled forms of motivation are moved by external sources and motives, are perceived as external to the self and are associated with higher rates of sport dropout [7].

SDT assumes that intrapersonal and interpersonal factors effect on athlete’s motivation [11]. The former are inherent in personal characteristics, such as gender, age, ethnicity and personality traits. The latter concern all social factors and experiences that influence personal motivation. For example, ample research has demonstrated how autonomous forms of motivation are more frequent in athletes or students who perceive autonomy support from their coaches or teachers, respectively [10,12]. Nevertheless, engagement and commitment can be influenced by extrinsic factors such as rewards, pressure from parents and coach, or intrinsic factors such as interest, desire for mastery and development [13,14].

Moreover, the satisfaction of an athlete’s basic psychological needs (autonomy, competence and relatedness) is largely influenced by the coach. Sport autonomy need is reinforced by a coach’s behavior style aimed at adopting strategies to support athlete’s autonomy, listening to their opinions and feelings, involving athletes in task planning and adopting adequate leadership styles. Competence need is supported by the coach’s behaviors aimed at planning achievable goals, implementing learning situations with clearly defined and communicated goals, using suitable feedback and reinforcing the athletes’ performance process over their results. Finally, relatedness need is supported by the coach’s actions to favor cooperation tasks, group dynamics, roles and responsibilities games among teammates [15,16,17].

Su and Reeve [18], in their meta-analysis of the effectiveness of intervention programs aimed to support autonomy in educational contexts, showed that autonomous behaviors were stimulated by coaching or teacher styles aimed at providing opportunities for personal initiatives and independent work, explanatory rationales for tasks and non-controlling language and acknowledging affects and emotions.

The framework of Achievement Goal Theory highlighted that individual goal orientations are influenced by the motivational climate such as a complex set of implicit and/or explicit environmental signs by the coach or teacher to outline success and failure events [2,3]. These goals were subdivided into ego- or performance-orientated goals and task- or mastery-orientated goals [2,3,19,20,21]. Athletes with ego-oriented goals perceive the performance as a way to assess their personal value when compared to others and, as a consequence, tend to demonstrate lower concern for “the process of competitive sport” [22] (p. 85). They tend to adopt achievement targets in order to ensure positive judgements of their skills, by adopting comparative and normative evaluations of their skills. Athletes with task-oriented goals perceive the performance as result of task mastery and represent skills as potentials that can be improved and increased by effort. As a consequence, they tend to master new abilities, thereby increasing their competence, adopting effective strategies, favoring challenging tasks and focusing more on effort [23]. Ego-oriented goals are reinforced by a performance climate where the coach’s most important goals are addressed to team wins, inter-individual competition and rivalry between team members. Such a climate is likely to generate anxiety regarding performance and reduce satisfaction with the sport’s environment [24], whereas task-oriented goals are reinforced by a mastery climate where the coach actively encourages learning through the correct treatment of errors, personal development, skill growth and cooperation between team members [25,26]. A mastery-orientated motivational climate is characterized by positive feelings such as enjoyment, satisfaction, interest, self-motivation and commitment, while a performance-orientated motivational climate is characterized by higher levels of anxiety and feelings of pressure. Enjoyment was demonstrated to be an important encouraging motive for physical activity behavior with positive influences on sport success performance [27,28,29].

Sport commitment has received less attention within the framework of SDT even though it plays a key role in explaining why an athlete is motivated to take part in sport. Sport commitment is composed of six dimensions: perceptions of the level of sport practice enjoyment, personal investments, opportunities of involvement, social restrictions, alternatives to involvement and social support [30,31,32]. Other models reinterpreted commitment as a bidimensional variable, composed of the athlete’s perception of effort in sport and intention to future practice because of considered variables such as opportunities of involvement, social restrictions and alternatives to involvement as well as the enjoyment resulting from the sport [33]. Nevertheless, sport commitment is a crucial variable to be considered in the interplay between the coach’s motivational climate and the athletes’ motivational disposition.

Based on these theoretical premises, the aim of the current study is examine the effects of the motivational climate created by the coach and perceived by a group of young high-performance handball players on their sport motivation, self-determination, sport psychological needs such as autonomy (feeling to have control over personal actions), competence (perception to master adequate ability) and relatedness (sense to belong to the group, coach or sport), and sport commitment. The participants’ age ranged from 16 to 17 years. The choice of the adolescence age was motivated by the crucial role played by the coach in this developmental phase. For adolescent athletes, the coach is a model who helps them to develop their autonomy, competence and relatedness through his behavior and instructions [33,34].

In detail, it was hypothesized that a perceived mastery-oriented climate was positively related to a higher level of autonomy and self-determined motivation, basic psychological needs satisfaction and higher sport commitment.

## 2. Materials and Methods

### 2.1. Participants

The design of this study was observational and descriptive of a transversal nature [35]. The sample included a total of 479 youth-category handball players (250 boys and 229 girls) selected to compete in the Spanish Regional Championships. These selected athletes are the best handball players in their regions. They are the regional selections and most of them have participated in more than two Spanish handball championships. These players are rated “high-performance athletes” by the Spanish Sports Council according to Royal Decree 971/2007, of 13 July, on high-level and high-performance athletes. The age range was 16 (40.1%)–17 (59.9%) years old (M = 16.60; SD = 0.50). With regard to the years of experience variable, 85.4% stated that they have over five years of experience at the federated handball player level.

### 2.2. Measurement Instruments

Perceived Motivational Climate in Sport Questionnaire (PMCSQ-2) [20,36]. The Spanish version of PMCSQ-2 was used [37,38]. The inventory includes 29 items grouped into two dimensions measuring the ego-involving (competitive) climate (14 items, e.g., “On this team, the coach gives most of his or her attention to the stars.”), and the task-involving (mastery) climate (15 items, e.g., “On this team, the coach emphasizes always trying to do your best”). Each item was headed with the phrase “In my training group or team…” Answers were collected on a Likert-type scale ranging from strongly disagree (1) to strongly agree (5). The alpha de Cronbach (α) was: mastery, α = 0.82; and competitive, α = 0.85.

Sport Motivation Scale (SMS) [39]. The original scale was called Échelle de Motivation dans les Sports (ÉMS) [40] and was translated to English by Pelletier et al. [39] with the name Sport Motivation Scale. The Spanish version of the SMS was used [41,42,43]. The scale has 28 items which assess the constructs of different motivation degrees established by the self-determination theory [1] suggesting a multidimensional explanation for motivation: amotivation (e.g., “I don’t know anymore; I have the impression that I am incapable of succeeding in this sport”), External Regulation (e.g., “because it allows me to be well regarded by people whom I know”), Introjected Regulation (e.g., “because it is absolutely necessary to do sports if one wants to be in shape”), Identified Regulation (e.g., “because, in my opinion, it is one of the best ways to meet people”), Intrinsic Motivation to Know (e.g., “for the pleasure it gives me to know more about the sport that I practice”), Intrinsic Motivation to Accomplishment (e.g., “for the pleasure I feel while improving some of my weak points”), and Intrinsic Motivation to Experience Stimulation (e.g., “for the pleasure I feel in living exciting experiences”). Each item was headed with the phrase “Why do you practice sport...” Answers were collected on a Likert-type scale, where options ranged from (1) does not correspond at all to (7) corresponds exactly; the average being (4) corresponds moderately.

In the present study, an overall index of self-determination motivation was used—the self-determination index (SDI). This index was created based on the ordered pattern of existing correlations in the motivational sub-scale as suggested by several authors (e.g., [44,45]). Several studies (e.g., [46,47]) have used this procedure. The SDI was calculated from the following expression: (((Intrinsic Motivation (IM) toward knowledge IM toward accomplishment IM toward experiencing stimulation)/3)2) Extrinsic Motivation (EM) identified regulation ((EM introjected regulation EM external regulation)/2)(Amotivation2)), as suggested by Vallerand [48].

Basic Psychological Needs in Exercise Scale (BPNES) [49]. The Spanish version of the BPNES was used [50]. The inventory was made up of 12 items grouped into three factors: autonomy (e.g., “the exercise programme I follow at the facility is in keeping with my interests”), competence (e.g., “I have made great progress as far as the result pursued is concerned), and relatedness (e.g., “I feel very comfortable when I do exercise with other participants”). This questionnaire used a Likert-type scale ranging from 1 (totally disagree) to 5 (totally agree). The internal consistency analysis was satisfactory (α = 0.84).

Sport Commitment Questionnaire (SCQ) [31]. The Spanish version of the SCQ was used [51]. The original scale has 28 items. The items are made up of 6 subscales: Sport Commitment has 6 items; Sport Enjoyment has 4 items; Personal Investments have 3 items; Social Constraints have 7 items; Involvement Opportunities and Involvement Alternatives have 4 items each. The answers were given on a 5-point Likert-type scale from strongly disagree (1) to strongly agree (5). “I like playing soccer this season” is an example of a Sport Enjoyment item. We presented items in an assertive/statement form instead of question form as presented by the original authors as Carpenter and Coleman [52] considered that it was easier for the participants to understand. The internal consistency analysis was satisfactory (α = 0.70).

Recently Scanlan, Chow, Sousa, Scanlan and Knifsend [53], have established psychometric properties of the Sport Commitment Questionnaire-2 (SCQ-2). This new instrument measures the updated Sport Commitment Model (SCM) [54] and replaces the obsolete SCQ, which evaluated the original SCM [31]. In Spain, Sánchez-Miguel et al. [55] have just adapted this scale to Spanish.

### 2.3. Procedure

The study was carried out during the Spanish Regional Championships (CESA). The Real Federación Española de Balonmano (RFEBM), the Federación Andaluza de Balonmano (FABM) and the coaches of the different regional selections all granted permission prior to our data collecting after reading a letter explaining the objectives of the study and the way it would be carried out. Prior to the administration of the questionnaires to the participants, and in accordance with the ethical guidelines of the American Psychological Association (APA), they were presented with an informed consent [35] for ethical compliance and data protection, ensuring, in this way, the rigor of the investigation and the privacy of the information obtained. The consent obtained from the players and their parents or tutors was both written and informed. A sample of the instrument was provided to them all. Data collection was carried out during the Spanish Championship, at the hotels where the teams were staying during players’ time off, in agreement with the coaches and in the presence of one of the researchers. Each participant had 20–30 min to complete the questionnaire and they were all briefed on the objects of the study and on their rights as participants, on the voluntary nature of the study and on the confidentiality of answers and data management. They were also informed that there were no correct or incorrect answers and were asked to give true and honest replies. Following data verification, the following variables were recorded: gender, year of birth, years of experience as a handball player, playing position, and the numbers of hours per week dedicated to training, as well as the times it was carried out. This study was carried out in accordance with the ethical guidelines of the American Psychological Association (APA). The protocol was approved by the Ethics Committee of the Universidad de Murcia (ID: 1494/2017). All subjects gave written informed consent in accordance with the Declaration of Helsinki [56].

### 2.4. Data Analysis

Means, standard deviation and bivariate correlations were analyzed for all variables under analysis. A two-step maximum likelihood (ML) approach suggested by Kline [57] in AMOS 23.0 (SPSS Inc., Chicago, IL, USA) was performed. Firstly, confirmatory factor analysis (CFA) was performed to analyze the psychometric properties of the purposed model. Composite reliability via Raykov [58] formula was performed to assess the internal consistency, taking 0.70 as the cut-off value [59], while the average variance extracted (AVE) was estimated to the analyzed convergent validity [59]. Discriminant validity was established when the correlation coefficients were lower than the AVE for each construct exceeding the squared correlations between that construct and any other construct [60]. Secondly, a structural equation model (SEM) was performed to test the proposed relationships among different constructs. For CFA and SEM, the following absolute and incremental indices were used for analysis: Comparative Fit Index (CFI), Normalized Fit Index (NFI), Standard Root Mean Residual (SRMR), and Root Mean Square Error of Approximation (RMSEA) with its Confidence Interval (CI: 90%). For these indices, scores of CFI and NFI ≥ 0.90, SRMR and RMSEA ≤ 0.08 were considered as acceptable, following several recommendations (e.g., [59,61,62]).

### 2.5. Mediation Analysis

For mediation analysis, the direct and indirect effects among constructs on outcome variable were analyzed as suggested by Hair et al. [59] and Hayes [63]. The bootstrap resampling procedure (1000 samples) via AMOS 23.0 was performed through bias corrected 95% confidence intervals (CI) was used to analyze the significance of direct and indirect effects. The indirect effect is considered significant (≤0.05) if its confidence interval does not include zero, as suggested by several authors (e.g., [63,64,65]). Effect sizes were evaluated as trivial (0–0.19), small (0.20–0.49), medium (0.50–0.79) and large (0.80 and greater), as suggested by Cohen [66].

## 3. Results

### 3.1. Preliminary Analyses

First, we performed CFAs to test the factorial structure of each scale. The findings showed that all scales (except the PMCSQ-2) exhibited acceptable to good fit indices with significant factor loadings. Because the CFA of the PMCSQ-2 showed unacceptable fit indices, we highlight the analyses of the PMCSQ-2. Taking into account the suggestion that authors should retain the subscale structure, but considering that a poor fit, we decided to exclude some items that have factor loadings less than 0.50 as suggest by Kline [57]. As a result, the PMCSQ-2 was composed of 10 items in the Task factor and seven items Ego factor.

A preliminary analysis revealed no missing values or outliers (univariate or multivariate). Data revealed also no violations from univariate distributions. However, Mardia’s coefficient form multivariate kurtosis exceeded the recommended value (>0.5). Thus, Bollen–Stine bootstrapping was performed [67]. Additionally, variance inflation factor (VIF) was calculated to check the collinearity diagnosis. Results indicate that all VIF values were less than 10, a recommended value suggested by Hair et al. [59]. Finally, GPower 3.1 (Heinrich-Heine-Universität Düsseldorf, Düsseldorf, Germany) [68] was used to determine the required sample size, considering the following parameters: (effect size f 2 = 0.1; a = 0.05; statistical power = 0.95 and four predictors) that the minimum required size would be 191 subjects, which was respected in the present study.

### 3.2. Measurement Model

Table 1 shows the means, standard deviations and bivariate correlations among variables. The athletes demonstrate high levels of a task-involving climate (M = 3.98; SD = 0.633), basic psychological needs satisfaction (M = 4.02; SD = 0.554), self-determined motivation (M = 7.35; SD = 3.80) and commitment (M = 4.32; SD = 0.605). On the contrary, the athletes show lower levels of an ego-involving climate (M = 2.71; SD = 0.737). The correlation patterns evidence that all constructs are positively and significantly associated between each other except the constructs related with an ego-involving climate. Finally, we observe that all constructs present adjusted values of composite reliability, all greater than or equal to 0.70 [59]. The test of the measurement model included task- and ego-involving climates, basic psychological needs satisfaction, self-determined index and commitment. Results show a good fit to the data (χ^2^ = 565.998 (290); SRMR = 0.052; B-Sp ≤ 0.001; RMSEA = 0.045 [90% CI = 0.039, 0.050]; NFI = 0.914; CFI = 0.923). Additionally, the measurement model revealed no problems of convergent and discriminant validity, since the average variance extracted was greater than or equal to 0.50 [59,60] and the square correlations among all constructs are less than the AVE of each factor [60].

### 3.3. Structural Model

**Figure 1:**
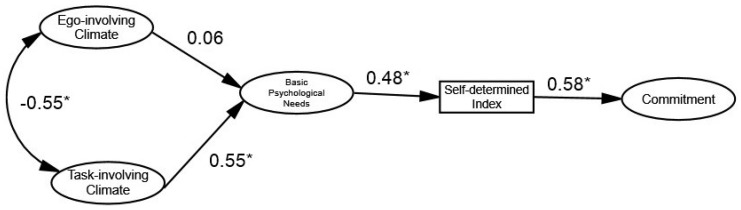
Standardized individual parameters—Hypothesized model. Note. (*) significant paths.

The structural model demonstrates a good fit to the data (χ^2^ = 619.004 (295); SRMR = 0.072; B-Sp ≤ 0.001; RMSEA = 0.048 [90% CI = 0.043, 0.053]; TLI = 0.90; CFI = 0.91). The standardized direct effect (Figure 1), positive and significant associations were observed among all constructs, except between an ego-involving climate and basic psychological needs satisfaction. Specifically, the association between a task-involving climate and basic psychological needs (β = 0.55 [0.387, 0.682]), basic psychological needs and self-determined index (β = 0.48 [0.376, 0.571]) and self-determined index and commitment (β = 0.58 [0.488, 0.663]) were all significant. Regarding the association between an ego-involving climate and basic psychological needs, it is not significant (β = 0.06 [−0.069, 0.181]).

Regarding mediation analysis between task- and ego-involving climates on self-determined index and commitment, as well as between basic psychological needs satisfaction on commitment, results show a positive and significant indirect effect among the aforementioned constructs, except between an ego-involving climate and self-determined index and sport commitment. Specially, the standardized indirect effect revealed that a task-involving climate positively predicted self-determined index (β = 0.27 [0.167, 0.363]) and commitment (β = 0.15 [0.091, 0.220]), via basic psychological needs satisfaction and self-determined index, respectively, and basic psychological needs positively predicted commitment (β = 0.28 [0.200, 0.363]). Furthermore, an ego-involving climate positively predicted both the self-determined index (β = 0.03 [−0.034, 0.085]) and commitment (β = 0.02 [−0.019, 0.050]), through needs satisfaction and the self-determined index, respectively. However, these indirect effects are not significant.

In sum, the mediation analysis revealed that basic psychological needs mediate the association between a task-involving climate and self-determined index, and self-determined index mediates the association between basic psychological needs satisfaction and commitment. Additionally, self-determined index mediates the association between a task-involved climate and commitment.

## 4. Discussion

The aim of this study was to examine the effects of the motivational climate created by the coach and perceived by a group of young high-performance handball players on their sport motivation, self-determination, sport psychological needs and sport commitment.

According to descriptive analyses, the handball players showed high levels of a task-involving climate and, conversely, lower levels of a ego-involving climate. Moreover, they demonstrated high basic psychological needs satisfaction, self-determined motivation and commitment. The correlation patterns evidenced that almost all investigated motivational constructs were positively and significantly associated. In more detail, higher levels of basic psychological needs such as autonomy (feeling to have control over personal actions), competence (perception to master adequate ability) and relatedness (sense to belong to the group, coach or sport) were associated with a higher task-involving climate, self-determined index and sport commitment; the self-determined index was related to commitment. This finding is coherent with research by other authors demonstrating how the motivational climate, generated by the coach, was closely related to his communication style, which effects a players’ sport commitment and enjoyment [69,70,71]. A motivational climate that rewarded effort and hard work as the main source of success is likely to generate a task orientation goal with satisfaction and proud of sport. Conversely, an extreme competitive-oriented climate is more likely to generate performance anxiety, reduced satisfaction with the sport and decreased athletes’ psychological well-being [25,26,72,73,74,75]. Balaguer et al. [25] demonstrated that handball players who perceived a task-oriented motivational climate showed positive performance and self-satisfaction with their performance improved. Moreover, their image of the coach was highly positive. While, handball players who perceived an ego-oriented motivational climate showed a negative image of their coach, even though they were satisfied of their team’s results.

As a concern mediation analysis, the obtained model showed that basic psychological needs mediated the association between a task-involving climate and self-determination, and self-determination mediated the association between basic psychological needs satisfaction and commitment. In detail, a task-involving climate positively predicted self-determined index and commitment, via basic psychological needs satisfaction and self-determined index, respectively; basic psychological needs positively predicted commitment. Furthermore, an ego-involving climate positively predicted both self-determined index and commitment, via needs satisfaction and self-determined index, respectively, but these indirect effects were not significant.

The important mediational role played by athletes’ basic psychological needs in sustaining self-determination and sport commitment is confirmed by much research [76,77]. The coach-created task climate was recognized to positively predict young players’ intrinsic motivation, future intention to play sport, persistence in sport, through autonomy, competence, relatedness, and intrinsic motivation.

Furthermore, basic psychological needs, such as autonomy (feeling to have control over personal actions), competence (perception to master adequate ability) and relatedness (sense to belong to the group, coach or sport), are predicted by athletes’ perceptions of autonomy supportive style and are non-oriented to control style [78].

A coach’s motivation climate that is aimed to adopt strategies to support athlete’s autonomy, listen to their opinions and feelings, plan achievable, reinforce athletes’ performance process over their results, is suitable to sustain athletes’ sport autonomy, competence and relatedness needs. Following self-determination theory, when psychological need satisfaction occurs, a personal sense of agency, effectiveness and belonging is generated in young athletes and this, in turn, favors sport engagement and commitment and prevents drop-out and maladaptive outcomes in youth sport [79,80]. Nevertheless, positive associations were demonstrated between need satisfaction and sport engagement, ranging from small/medium to large effects [81,82]. Moreover, sport commitment was enhanced and supported by high self-determination [83]. Lukwu and Guzmán [33] considered commitment as a bidimensional variable, influenced by the athlete’s perception of effort in sport and their intention of the future. They raised interesting explanations in adolescence. The authors underlined that in adolescence age, the coach is an important model and acts through his behavior and instructions to make adolescents more confident with their ability, friends in sport tasks, aware of the most relevant reasons to practice sport, and more engaged. At early age stages, results show that players’ parents and family have the strongest influence on children’s involvement in sports practice and on the creation of sports habits [84,85]. An increase in peer influence is observed during the first stage of adolescence, given that they provide support and social recognition and this role is shared with the figure of the coach as the main agent of influence through the motivational climate perceived in the sports environment [86]. All these factors, in turn, tend to increase youth sport commitment. With increasing age, peer influence, especially involving players of the same gender, gradually increases throughout adolescence and even exceeds that of parents and coaches [35,87,88].

More specifically, in the case of a team sport, such as handball, and at the height of adolescence, peers and coaches are the main social agents in a team [89] and they play a central role when it comes to achieving sporting excellence [24,73,90,91]; they are responsible for promoting a certain type of climate during training and thus affect the way players face the tasks proposed.

While, in adult age, the coach-created mastery climate is less effective to increase motivation and commitment because the coach is not considered an important model anymore. Its effect on motivation and commitment is probably only indirect and mediated by the satisfaction of psychological needs.

The developmental trajectory of relationships between motivational factors and sport commitment is an important question to be considered in future research. However, this is, at the same time, the strength of this research. To our knowledge, little research is focused on the links among coach-created climates, basic needs satisfaction, self-determination and sport commitment. This study, therefore, helps address important intervention implications as the implementation of training coaches to enhance athletes’ commitment by the satisfaction of motivational needs. These intervention programs need to focus on strategies to promote goal setting, needs satisfaction, e.g., by encouraging athletes to make their own decisions, communicate their feelings, participate in team and peer mentoring activities, participate in their assessment and become more aware of their engagement.

Finally, as limitations of the study, it should be mentioned that although the results extend the information provided in similar studies, the specificity of the sample examined limits its generalization. These results require confirmation in future research with individual athletes where the interaction with the coach is different. Further topics that could be addressed in the future are the influence on players of parents and peers, and the difference between an athlete’s and coach’s own perception of the coach-created motivational climate.

## Figures and Tables

**Table 1:** Descriptive and correlation analysis for all constructs and composite reliability.

Constructs	TI	EI	BPN	SDI	Commitment
Task involving	1	-	-	-	-
Ego involving	−0.385 **	1	-	-	-
BPN	0.426 **	−0.140 **	1	-	-
SDI	0.330 **	−0.275 **	0.405 **	1	-
Commitment	0.301 **	−0.141 **	0.454 **	0.473 **	1
Mean	3.98	2.71	4.02	7.35	4.32
SD	0.633	0.737	0.554	3.80	0.605
Range	2–5	1–5	2–5	−5.79–16.13	2–5
CR	0.82	0.86	0.77	-	0.70

Note. TI = task-involving climate; EI = ego-involving climate; BPN = basic psychological needs (composite factor); SDI = self-determined index); CR = composite reliability; SD = standard deviation; ** *p* ≤ 0.01.

## References

[1] Deci E.L., Ryan R.M. (1985). Intrinsic Motivation and Self-Determination in Human Behaviour.

[2] Ames C., Roberts G.C. (1992). Achievement goals, motivational climate, and motivational processes. Motivation in Sport and Exercise.

[3] Nicholls J.G. (1989). The Competitive Ethos and Democratic Education.

[4] Alesi M., Bianco A., Padulo J., Luppina G., Petrucci M., Paoli A., Palma A., Pepi A. (2015). Motor and cognitive growth following a Football Training Program. Front. Psychol..

[5] Deci E.L., Vallerand R.J., Pelletier L.G., Ryan R.M. (1991). Motivation and education: The self-determination perspective. Educ. Psychol..

[6] Moè A., Katz I., Alesi M. (2018). Scaffolding for motivation by parents, and child homework motivations and emotions: Effects of a training programme. Brit. J. Educ. Psychol..

[7] Ryan R.M., Deci E.L. (2017). Self-Determination Theory: Basic Psychological Needs in Motivation, Development, and Wellness.

[8] Deci E.L., Ryan R.M. (1985). The general causality orientations scale: Self-determination in personality. J. Res. Pers..

[9] Ryan R.M., Deci E.L. (2000). Self-determination theory and the facilitation of intrinsic motivation, social development, and well-being. Am. Psychol..

[10] Pelletier L.G., Fortier M.S., Vallerand R.J., Brière N.M. (2001). Associations among perceived autonomy support, forms of self-regulation, and persistence: A prospective study. Motiv. Emotion..

[11] Deci E.L., Ryan R.M. (2008). Facilitating optimal motivation and psychological well-being across life’s domains. Can. Psychol..

[12] Hagger M.S., Chatzisarantis N.L.D., Culverhouse T., Biddle S.J.H. (2003). The process by which perceived autonomy support in physical education promotes leisure-time physical activity intentions and behavior: A trans-contextual model. J. Educ. Psychol..

[13] Pelletier L.G., Rocchi M.A., Vallerand R.J., Deci E.L., Ryan R.M. (2013). Validation of the revised sport motivation scale (SMS-II). Psychol. Sport Exerc..

[14] Vallerand R.J., Tennenbaum G., Eklund R.C. (2007). Intrinsic and extrinsic motivation in sport and physical activity: A review and a look at the future. Handbook of Sport Psychology.

[15] Fenton S.A., Duda J.L., Quested E., Barrett T. (2014). Coach autonomy support predicts autonomous motivation and daily moderate-to-vigorous physical activity and sedentary time in youth sport participants. Psychol. Sport Exerc..

[16] Jang H., Reeve J., Deci E.L. (2010). Engaging students in learning activities: It is not autonomy support or structure but autonomy support and structure. J. Educ. Psychol..

[17] Solstad B.E., van Hoye A., Ommundsen Y. (2015). Social contextual and intrapersonal antecedents of coaches’ basic need satisfaction: The intervening variable effect of providing autonomy-supportive coaching. Psychol. Sport Exerc..

[18] Su Y.L., Reeve J.A. (2011). A meta-analysis of the effectiveness of intervention programs designed to support autonomy. Educ. Psychol. Rev..

[19] Gómez-López M., Granero-Gallegos A., Isorna M. (2013). Analysis of psychological factors that affect high athletic performance in kayakers. Rev. Iberoam. Diagn. Eval..

[20] Newton M., Duda J.L., Yin Z. (2000). Examination of the psychometric properties of the Perceived Motivational Climate in Sport Questionnaire-2 in a simple of female athletes. J. Sport Sci..

[21] Seifriz J.J., Duda J.L., Chi L. (1992). The relationship of perceived motivational climate and intrinsic motivation and beliefs about success in basketball. J. Sport Exerc. Psychol..

[22] Duda J.L., Olson L.K., Templin T.J. (1991). The relationship of task and ego orientation to sportsmanship attitudes and the perceived legitimacy of injurious acts. Res. Q. Exerc. Sport..

[23] Pepi A., Alesi M., Pecoraro D., Faria L. (2015). Incremental-entity personal conceptions of intelligence and individualism-collectivism in Italian students. MJSS.

[24] Granero-Gallegos A., Gómez-López M., Rodríguez-Suárez N., Abraldes J.A., Alesi M., Bianco A. (2017). Importance of the Motivational Climate in Goal, Enjoyment, and the Causes of Success in Handball Players. Front. Psychol..

[25] Balaguer I., Duda J.L., Atienza F.L., Mayo C. (2002). Situational and dispositional goals as predictors of perceptions of individual and team improvement, satisfaction and coach ratings among elite female handball teams. Psychol. Sport Exerc..

[26] Pensgaard A.M., Roberts G.C. (2000). The relationship between motivational climate, perceived ability and sources of distress among elite athletes. J. Sport Sci..

[27] Dishman R.K., Motl R.W., Saunders R., Felton G., Ward D.S., Dowda M., Pate R.R. (2005). Enjoyment mediates the effects of a school-based physical activity intervention among adolescent girls. Med. Sci. Sports Exerc..

[28] Hashim H., Grove J.R., Whipp P. (2008). Validating the youth sport enjoyment construct in high school physical education. Res. Q. Exerc. Sport..

[29] Timo J., Sami Y.P., Anthony W., Jarmo L. (2016). Perceived physical competence towards physical activity, and motivation and enjoyment in physical education as longitudinal predictors of adolescents’ self-reported physical activity. J. Sci. Med. Sport..

[30] Scanlan T.K., Carpenter P.J., Schmidt G.W., Simons J.P., Keeler B. (1993). An introduction to the sport commitment model. J. Sport Exerc. Psychol..

[31] Scanlan T.K., Simons J.P., Carpenter P.J., Schmidt G.W., Keeler B. (1993). The sport commitment model: Measurement development for the youth-sport domain. J. Sport Exerc. Psychol..

[32] Scanlan T.K., Russell D.G., Beals K.P., Scanlan L.A. (2003). Project on elite athlete commitment (PEAK): II. A direct test and expansion of the sport commitment model with elite amateur sportsmen. J. Sport Exerc. Psy..

[33] Lukwu R.M., Guzmán J.F. (2011). Sport commitment and adherence: A social-cognitive analysis. RICYDE.

[34] Méndez-Giménez A., Fernández-Río J., Cecchini J.A. (2012). Student’ important role, basic psychological needs, motivational regulations, and physical self-concept in physical education setting. CPD.

[35] Gómez-López M., Ruiz-Sánchez V., Granero-Gallegos A. (2019). Analysis of the Prediction of Motivational Climate in Handball Players’ Fear of Failure. Int. J. Environ. Res. Public Health.

[36] Walling M.D., Duda J.L., Chi L. (1993). The perceived motivational climate in sport questionnaire: Construct and predictive validity. J. Sport Exerc. Psychol..

[37] Balaguer I., Givernau M., Duda J.L., Crespo M. (1997). Analysis of the validity of the construct and the predictive validity of the questionnaire of motivational climate perceived in sport (PMCSQ-2) with Spanish competition tennis players. Rev. Psicol. Deporte.

[38] Balaguer I., Mayo C., Atienza F.L., Duda J.L. (1997). Factorial validity of the Perceived Motivational climate in Sport Questionnaire-2 in the case of Spanish elite female handball teams [Abstract]. J. Sport Exerc. Psychol..

[39] Pelletier L.G., Fortier M.S., Vallerand R.J., Tuson K.M., Brière N.M., Blais M.R. (1995). Toward a new measure of intrinsic motivation, extrinsic motivation, and a motivation in sports: The Sport Motivation Scale (SMS). J. Sport Exerc. Psychol..

[40] Brière N., Vallerand R., Blais N., Pelletier L. (1995). Development and validation of a scale on intrinsic and extrinsic motivation and lack of motivation in sports: The Scale on Motivation in Sports. Int. J. Sport Psychol..

[41] Balaguer I., Castillo I., Duda J.L. (2007). Psychometric properties of the sports motivation scale in Spanish athletes. Rev. Mex. Psicol..

[42] Núñez J.L., Martín-Albo J., Navarro J.G., González V.M. (2006). Preliminary Validation of a Spanish Version of the Sport Motivation Scale. Percept. Motor Skills.

[43] Núñez J.L., Martín-Albo J., Navarro J.G. (2007). Psychometric properties of the Spanish version of the sport motivation scale. Rev. Psicol. Deporte.

[44] Ryan R., Connell J. (1989). Perceived locus of causality and internalization: Examining reasons for acting in two domains. J. Pers. Soc. Psychol..

[45] Howard J., Gagné M., Bureau J. (2017). Testing a continuum structure of self-determined motivation: A meta-analysis. Psychol. Bull..

[46] Sarrazin P., Vallerand R., Guillet E., Pelletier L., Cury F. (2002). Motivation and dropout in female handballers: A 21-month prospective study. Eur. J. Soc. Psychol..

[47] Guzmán J., Kingston K. (2012). Prospective Study of Sport Dropout: A motivational analysis as a function of age and gender. Eur. J. Sport Sci..

[48] Vallerand R., Zanna M.P. (1997). Toward a hierarchical model of intrinsic and extrinsic motivation. Advances in Experimental Social Psychology.

[49] Vlachopoulos S.P., Michailidou S. (2006). Development and initial validation of a measure of autonomy, competence, and relatedness in exercise: The Basic Psychological Needs in Exercise Scale. MPEES.

[50] Sánchez J.M., Núñez J.L. (2007). Preliminary analysis of the psychometric properties of the Spanish version of the Scale of Basic Psychological Needs in Physical Exercise. RIPED.

[51] Sousa C., Torregrosa M., Viladrich C., Villamarín F., Cruz J. (2007). The commitment of young soccer players. Psicothema.

[52] Carpenter P.J., Coleman R. (1998). A longitudinal study of elite youth cricketers’ commitment. Int. J. Sport Psychol..

[53] Scanlan T., Chow G., Sousa C., Scanlan L.A., Knifsend C.A. (2016). The development of the Sport Commitment Questionnaire-2 (English version). Psychol. Sport Exerc..

[54] Scanlan T., Russell D., Scanlan L., Klunchoo T., Chow G. (2013). Project on Elite Athlete Commitment (PEAK): IV. Identification of New Candidate Commitment Sources in the Sport Commitment Model. J. Sport Exerc. Psychol..

[55] Sánchez-Miguel P.A., Chow G.M., Sousa C3 Scanlan T.K., Ponseti F.J., Scanlan L., García-Mas A. (2019). Adapting the Sport Commitment Questionnaire-2 for Spanish Usage. Percept. Motor Skills.

[56] World Medical Association (2013). World Medical Association Declaration of Helsinki: Ethical Principles for Medical Research Involving Human Subjects. JAMA.

[57] Kline R. (2016). Principles and Practice of Structural Equation Modelling.

[58] Raykov T. (1997). Estimation of composite reliability for congeneric measures. Appl. Psych. Meas..

[59] Hair J., Black W., Babin B., Anderson R. (2014). Multivariate Data Analysis.

[60] Fornell C., Larcker D.F. (1981). Evaluating structural equation models with unobservable variables and measurement error. J. Mark. Res..

[61] Byrne B. (2010). Structural Equation Modeling with AMOS Basic Concepts, Applications, and Programming.

[62] Marsh H., Hau K., Wen Z. (2004). In search of golden rules: Comment on hypothesis testing approaches to setting cutoff values for fit indexes and dangers in overgeneralizing Hu and Bentler’s (1999) findings. Struct. Equ. Model..

[63] Hayes A.F. (2018). Introduction to Mediation, Moderation, and Conditional Process Analysis: A Regression-Based Approach.

[64] Mackinnon D.P., Lockwood C.M., Williams J. (2004). Confidence Limits for the Indirect Effect: Distribution of the Product and Resampling Methods. Multivar. Behav. Res..

[65] Williams J., MacKinnon D.P. (2008). Resampling and distribution of product methods for testing indirect effects in complex models. Struct. Equ. Model..

[66] Cohen J. (1992). A power primer. Psychol. Bull..

[67] Nevitt J., Hancock G.R. (2001). Performance of bootstrapping approaches to model test statistics and parameter standard error estimation in structural equation modeling. Struct. Equ. Model..

[68] Faul F., Erdfelder E., Buchner A., Lang A.G. (2009). Statistical power analyses using G*Power 3.1: Tests for correlation and regression analyses. Behav. Res. Methods.

[69] Torregrosa M., Sousa C., Viladrich C., Villamarín F., Cruz J. (2008). Motivational climate and coaches’ communication style predict young soccer players’ commitment. Psicothema.

[70] Moreno J.A., Cervelló E., González-Cutre D. (2007). Young athletes’ motivational profiles. J. Sport Sci. Med..

[71] Murillo M., Sevil J., Abós Á., Samper J., Abarca-Sos A., García-González L. (2018). Analysis of the sport commitment of young waterpolists: A study grounded in self-determination theory. RIPED.

[72] Duda J.L., Roberts G.C. (2001). Achievement Goal Research in Sport: Pushing the Boundaries and Clarifying Some Misunderstandings. Advances in Motivation in Sport and Exercise.

[73] Gómez-López M., Granero-Gallegos A., Baena-Extremera A., Abraldes J.A. (2014). Goal orientation effects on elite handball players motivation and motivational climate. Procedia Soc. Behav. Sci..

[74] Ruiz-Juan F., Gómez-López M., Pappous A., Alacid F., Flores G. (2010). Dispositional goal orientation, beliefs about the causes of success and intrinsic satisfaction in young elite paddlers. J. Hum. Kinet..

[75] Vazou S., Ntoumanis N., Duda J.L. (2006). Predicting young athletes’ motivational indices as a function of their perceptions of the coach- and peer-created climate. Psychol. Sport Exerc..

[76] Alvarez M.S., Balaguer I., Castillo I., Duda J.L. (2012). The coach-created motivational climate, young athletes’ well-being, and intentions to continue participation. JCSP.

[77] Balaguer I., González L., Fabra P., Castillo I., Mercé J., Duda J.L. (2012). Coaches’ interpersonal style, basic psychological needs and the well- and ill-being of young soccer players: A longitudinal analysis. J. Sport Sci..

[78] González L., Tomás I., Castillo I., Duda J.L., Balaguer I. (2017). A test of basic psychological needs theory in young soccer players: Time-lagged design at the individual and team levels. Scand. J. Med. Sci. Sports.

[79] Bartholomew K.J., Ntoumanis N., Ryan R.M., Thøgersen-Ntoumani C. (2011). Psychological need thwarting in the sport context: Assessing the darker side of athletic experience. J. Sport Exerc. Psychol..

[80] Jowett G.E., Hill A.P., Hall H.K., Curran T. (2016). Perfectionism, burnout and engagement in youth sport: The mediating role of basic psychological needs. Psychol. Sport Exerc..

[81] De Francisco C., Arce C., Sánchez-Romero E.I., Vílchez M. (2018). The mediating role of sport self-motivation between basic psychological needs satisfaction and athlete engagement. Psicothema.

[82] Podlog L., Gustafsson H., Skoog T., Gao Z., Westin M., Werner S., Alricsson M. (2015). Need satisfaction, motivation, and engagement among high-performance youth athletes: A multiple mediation analysis. IJSEP.

[83] Zahariadis P., Tsorbatzoudis H., Alexandris K. (2006). Self-Determination in Sport Commitment. Percept. Motor Skills.

[84] Duda J.L., Ntoumanis N., Mahoney J.L., Larson R.W., Eccles J.S. (2005). After-School Sport for Children: Implications of a Task-Involving Motivational Climate.

[85] Duda J.L., Papaioannou A.G., Appleton P.R., Quested E., Krommidas C., Papaioannou A.G., Hackfort D. (2014). Creating adaptive motivational climates in sport and physical education. Routledge Companion to Sport and Exercise Psychology: Global Perspectives and Fundamental Concepts.

[86] Ntoumanis N., Vazou S., Duda J.L., Jowette S., Lavallee D. (2007). Peer-created motivational climate. Social Psychology in Sport.

[87] Castillo I., Balaguer I., Duda J.L., García-Merita M.L. (2004). Psychosocial factors associated with sports practice during adolescence. Rev. Latinoam. Am. Psicol..

[88] Moreno-Murcia J.A., Conte L. (2011). Prediction of fear to err in basketball players through the peer motivational climate and intrinsic motivation. Rev. Mex. Psicol..

[89] Duda J.L., Balaguer I., Jowette S., Lavallee D. (2007). The coach-created motivational climate. Social Psychology in Sport.

[90] Granero-Gallegos A., Gómez-López M., Baena-Extremera A., Abraldes J.A., Rodríguez-Suárez N. (2012). Self-determined motivation in amateur handball. Rev. Iberoam. Diagn. Eval. Psicol..

[91] Ortiz P., Gómez-López M., Martín I., Reigal R.E., García-Mas A., Chirosa L.J. (2016). Role played by the coach in the adolescent players’ commitment. Stud. Psychol..

